# Precocious Puberty or Premature Thelarche: Analysis of a Large Patient Series in a Single Tertiary Center with Special Emphasis on 6- to 8-Year-Old Girls

**DOI:** 10.3389/fendo.2017.00213

**Published:** 2017-08-23

**Authors:** Tero Varimo, Heta Huttunen, Päivi Johanna Miettinen, Laura Kariola, Johanna Hietamäki, Annika Tarkkanen, Matti Hero, Taneli Raivio

**Affiliations:** ^1^Children’s Hospital, Pediatric Research Center, University of Helsinki, Helsinki University Hospital, Helsinki, Finland; ^2^Faculty of Medicine, Department of Physiology, University of Helsinki, Helsinki, Finland; ^3^Research Programs Unit, Molecular Neurology, Biomedicum Stem Cell Center, University of Helsinki, Helsinki, Finland

**Keywords:** precocious puberty, premature thelarche, growth velocity, brain MRI, predictors of puberty

## Abstract

**Introduction:**

We describe the etiology, MRI findings, and growth patterns in girls who had presented with signs of precocious puberty (PP), i.e., premature breast development or early menarche. Special attention was paid to the diagnostic findings in 6- to 8-year-olds.

**Materials and methods:**

We reviewed the medical records of 149 girls (aged 0.7–10.3 years) who had been evaluated for PP in the Helsinki University Hospital between 2001 and 2014.

**Results:**

In 6- to 8-year-old girls, PP was most frequently caused by idiopathic gonadotropin-releasing hormone (GnRH)-dependent PP (60%) and premature thelarche (PT; 39%). The former subgroup grew faster (8.7 ± 2.0 cm/year, *n* = 58) than the girls with PT (7.0 ± 1.1 cm/year, *n* = 32) (*P* < 0.001), and the best discrimination for GnRH-dependent PP was achieved with a growth velocity cut-off value of 7.0 cm/year (sensitivity 92% and specificity 58%) [area under the curve 0.82, 95% confidence interval (CI) 0.73–0.91, *P* < 0.001]. Among asymptomatic and previously healthy 6- to 8-year-old girls with GnRH-dependent PP, one (1.7%, 95% CI 0.3–9.7%) had a pathological brain MRI finding requiring surgical intervention (craniopharyngioma). In girls younger than 3 years, the most frequent cause of breast development was PT, and, in 3- to 6-year-olds, GnRH-dependent PP.

**Conclusion:**

In 6- to 8-year-old girls, analysis of growth velocity is helpful in differentiating between PT and GnRH-dependent PP. Although the frequency of clinically relevant intracranial findings in previously healthy, asymptomatic 6- to 8-year-old girls was low, they can present without any signs or symptoms, which favors routine MRI imaging also in this age group.

## Introduction

During the last 50 years, there has been a continuing downward trend toward an earlier timing of puberty, evidenced in girls by an earlier mean age of breast development and, to lesser extent, menarche (1, 2). In girls, puberty is considered to be precocious when it occurs before the age of 8 years (3–5), and when precocious puberty (PP) manifests secondary to the activation of the hypothalamic–pituitary–gonadal (HPG) axis, the condition is considered to be gonadotropin-releasing hormone (GnRH) dependent. However, secondary sexual characteristics can occur without the activation of the HPG axis. For example, the premature appearance of glandular breast tissue or pubic hair can be caused by benign variants premature thelarche (PT) or adrenarche, or by pathologic GnRH-independent PP such as in McCune–Albright syndrome or in congenital adrenal hyperplasia (6).

GnRH-dependent PP is a relatively rare condition showing an incidence of approximately 0.2% (7), and in girls approximately 95% of the cases are idiopathic (8–10). In the remaining proportion, the premature activation of the HPG axis is caused by an abnormality in the central nervous system (CNS), which emphasizes the need to identify these subjects as early as possible. At the same time, incidental findings in brain MRI are relatively common and their role in GnRH-dependent PP are not always clear (10, 11). Young age is a major predictor of CNS pathology, whereas the prevalence of pathologic GnRH-dependent PP declines with age being approximately 2% in girls above 6 years according to previous reports (8, 10). Therefore, the Lawson Wilkins Pediatric Endocrine Society (LWPES) and European Society of Pediatric Endocrinology (ESPE) recommended that in girls with GnRH-dependent PP, a brain MRI should be performed in all subjects less than 6 years of age; whereas in girls aged 6–8 years, brain MRI may be reserved to those who show a rapid progression of puberty or neurologic signs or symptoms (12). The suggested guideline has been supported by some studies (10, 13, 14), whereas others have questioned it, and currently no consensus or evidence-based criteria for brain imaging exist for 6- to 8-year-old girls (9, 11, 12).

The prevalence of PT has increased in girls younger than 8 years without a parallel increase in estradiol or gonadotropin levels (15, 16). Thus, identifying girls with PT from those with GnRH-dependent PP may be challenging especially among the younger girls (17, 18). Increased growth velocity serves as a good indirect marker of estrogen activity (through GH/IGF-1 axis activation) (19), but its clinical use in the differential diagnosis of PP is, still, an open-ended question.

The aim of this study was to report the etiology of PP in a tertiary center with special focus on the imaging findings of girls with GnRH-dependent PP who were diagnosed between the ages of 6 and 8 years. In addition, we evaluated if growth velocity could effectively differentiate between PT and GnRH-dependent PP.

## Materials and Methods

**Figure 1:**
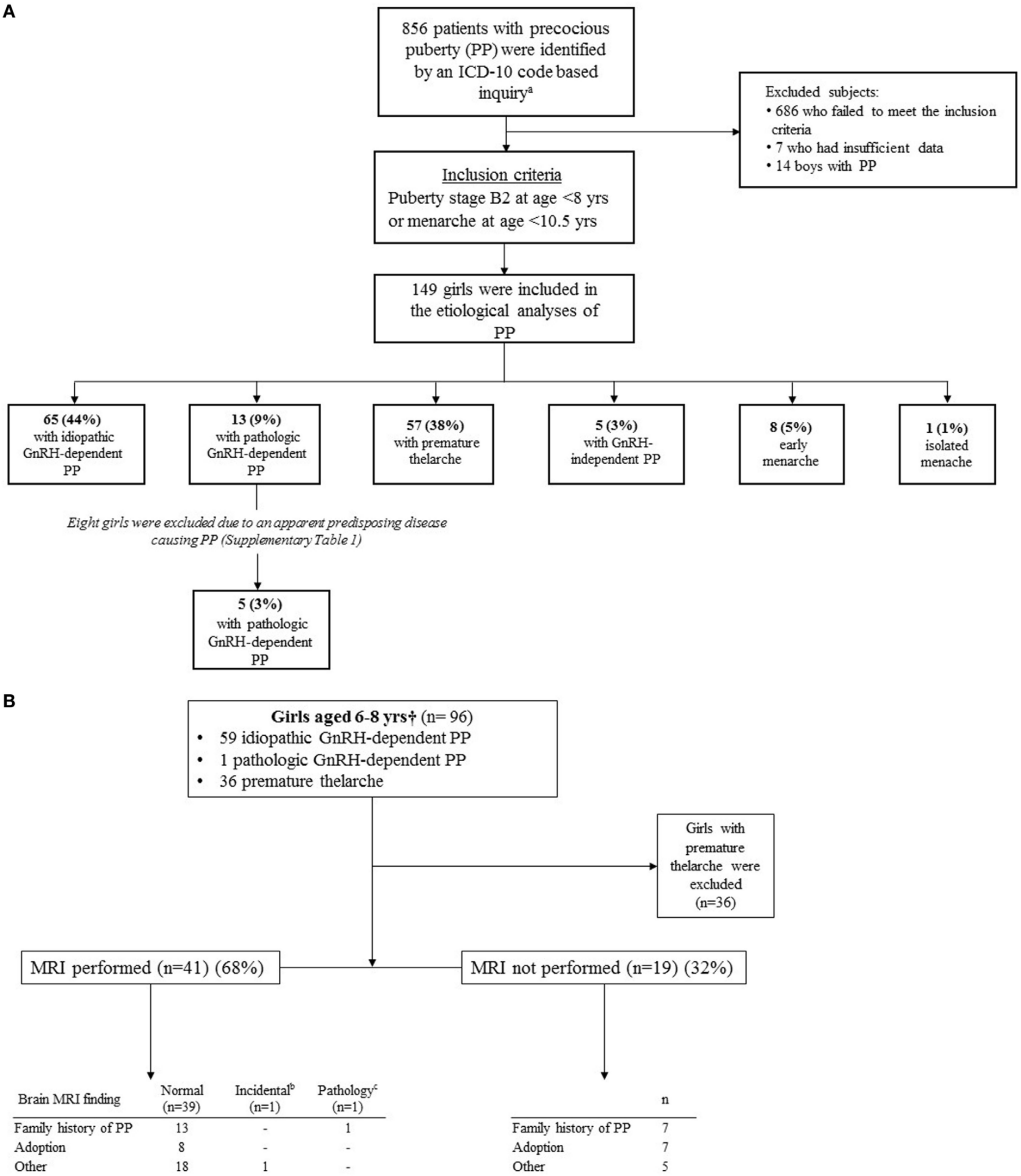
**(A)** Enrollment and outcomes of 149 girls with precocious puberty (PP). **(B)** Underlying causes of PP and brain MRI findings in 96 girls aged 6–8 years. ^a^ICD-10 codes: E22.80, E28, E28.8, E28.9, E29, E29.0, E29.8, E29.9, E30, E30.1, E30.8, E30.9, and Q78.1. ^b^Pineal cyst. ^c^Craniopharyngioma. ^†^Girls aged 6–8 years without a disease that predisposes to PP.

An ICD-10 code-based inquiry was performed to the electronic patient records to identify patients who were evaluated for signs of PP in the Pediatric Endocrine Outpatient Clinic of Helsinki University Hospital (HUH) between 2001 and 2014 (Figure 1). The following ICD-10 codes were used: E22.80, E28, E28.8, E28.9, E29, E29.0, E29.8, E29.9, E30, E30.1, E30.8, E30.9, and Q78.1. The inquiry resulted in 856 patients. Of them, 149 girls fulfilled the eligibility criteria of PP: clinical signs of puberty defined as Tanner breast stage 2 before age 8 or menarche at the age of 10.3 years or younger (i.e., the mean −3 SD of healthy Finnish girls) (3, 5). The ethnicities of the girls were as follows: 126 (85%) Caucasian, 12 (8%) African, 5 (3%) Asian, 4 (3%) Asian-Chinese, and 2 (1%) Hispanic. Subsequently, the girls were categorized into six diagnostic subgroups based on the hormonal, clinical, and imagining findings: PT, early menarche, isolated menarche, GnRH-independent PP, idiopathic GnRH-dependent PP, and pathologic GnRH-dependent PP (20) (Table 1; the cut-off levels of gonadotropins are provided in the footnote). These girls formed the population that was used to describe the etiology of PP (Figure 1A). Ten girls were previously diagnosed with a disease known to predispose to PP (Table S1 in Supplementary Material), and the remaining girls (*n* = 139) without a known predisposing disease were included in the further analysis (Figure 1A). Of them, subjects aged 6–8 years (*n* = 96) were analyzed as a separate subgroup (Figure 1B). Since the focus of the study was in the girls with PP, 14 boys with PP, although identified by the inquiry, were excluded from the series (Figure 1A).

**Table 1:** Classification of girls presenting with signs of precocious puberty.

Diagnosis	Criteria
Premature thelarche	B2–5 less than 8 years of age and prepubertal LH^a^
Early menarche	Menarche at the age of 10.3 years or younger and B2 or more
Isolated menarche	As above, but B1
GnRH-independent precocious puberty	B2–5 less than 8 years of age, elevated estradiol, and prepubertal LH
GnRH-dependent precocious puberty	B2–5 less than 8 years of age and pubertal LH^b^
Pathologic GnRH-dependent precocious puberty	As above, plus an abnormal brain MRI finding

*^a^Peak serum LH ≤ 5 IU/L in a GnRH stimulation test or basal serum LH < 0.3 IU/L*.

*^b^Peak serum LH > 5 IU/L in a GnRH stimulation test or basal serum LH ≥ 0.3 IU/L. In girls younger than 3 years, documented progression of puberty or an abnormal brain MRI (21)*.

*B, Tanner breast stage*.

The family history of PP was considered positive if a first degree relative had had Tanner breast stage 2 before the age of 8 years or menarche before the age of 11 years or if the father reported a very early timing of puberty. The information was available in all girls and it was assessed by a pediatric endocrinologist. Gonadotropin and estradiol levels were obtained and measured as described before (22) and were available as follows: LH in 143 (96%), FSH in 142 (95%), GnRH stimulation test in 120 (81%), and estradiol in 136 (91%).

Growth data were collected in girls older than 3 years and were available in 120 (97%) girls (60 with idiopathic GnRH-dependent PP, 39 with PT, 11 with pathologic GnRH-dependent PP, 9 with early or isolated menarche, and one with GnRH-independent PP). Height and weight were measured by trained nurses in maternity hospitals, child welfare clinics, school health care, and the university hospital endocrinology clinic as described previously in detail (23). First, the growth curves were visually assessed to exclude obvious measuring errors. Then the growth preceding the initial evaluation of PP was modeled by determining growth between the initial evaluation and the point 6–24 months before the initial evaluation. Growth velocity and an annual change in height SDS between the two time points were calculated. In subjects with annual change in height SDS exceeding +0.3 SD, the age at pubertal take-off (i.e., the nearest point in the growth curve that precedes the increase in growth velocity) was identified (24). Finally, weight-for-height and BMI values were used to describe weight progression (25, 26).

This study was approved by the HUH and the ethics committee for gynecology and obstetrics, pediatrics, and psychiatry of HUH.

### Statistical Analyses

The data are presented with number (percentage) or mean ± SD. SPSS statistical software version 22.0 (SPSS, Chicago, IL, USA) was used in statistical analyses. Between-group comparisons were performed with *T*- and chi-square-test, and Spearman’s rank correlation was used in correlation analysis. In 6- to 8-year-old girls, linear regression analysis was used in describing the changes in number of subjects with PT during the study period, whereas logistic regression analysis was used to study the associations of clinical measurements with the odds of GnRH-dependent PP. Age is a known contributor to the onset of puberty (8); thus, the logistic regression analyses were run univariate and adjusted for age. The results are shown as odds ratios and 95% confidence intervals (CIs). In addition, the diagnostic performance of growth velocity in the differential diagnostics between GnRH-dependent PP and PT was evaluated by constructing a receiver operating curve (ROC). Area under the curve (AUC) with 95% CIs were calculated from ROC. The cut-off value that maximizes sensitivity and specificity was selected. All tests were two-sided and the statistical significant level was set to *P* < 0.05.

## Results

**Figure 2:**
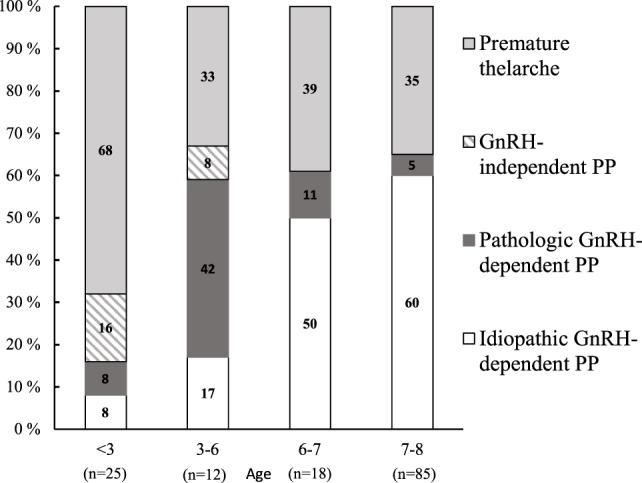
The distribution of causes that underlie precocious puberty (PP) in girls aged 0.7–8 years.

The age-dependent etiologies of PP in girls are shown in Figure 2. In 6- to 8-year-old girls, the annual number of patients with PT increased during the study period (*r* = 0.6, *P* = 0.03), and the frequency of patients with PT showed similar trend but did not reach statistical significance (*r* = 0.5, *P* = 0.09). At the same time, the number of subjects with idiopathic GnRH-dependent PP remained constant (*r* = 0.2, *P* = 0.50). PT explained almost 70% of cases with PP aged less than 3 years (Figure 2). In the older girls (3–6 years of age), PT explained only 33% of cases with PP, whereas GnRH-dependent PP was now the most common etiology (59%). In the latter age group, pathological GnRH-dependent PP was more frequent than the idiopathic form, and the frequency of pathologic GnRH-dependent PP was higher than in girls aged 6–8 years (χ^2^ = 16.2, *P* < 0.001). Overall, 67 (86%) girls with GnRH-dependent PP were treated with GnRH-analogs, including all girls presenting signs of PP at age less than 6 years, 10 girls (83%) presenting at age 6–7 years, and 46 girls (83%) presenting at age 7–8 years. Of note, 17 girls were older than 8 years at the initiation of GnRH-analog treatment. The vast majority of cases with GnRH-independent PP (4 out of 5) were diagnosed prior to the age of 3 years. The etiology of GnRH-independent PP was McCune–Albright syndrome in four girls and an ovarian cyst in one girl.

A positive family history of PP was found in 38 (25%) girls: 21 with idiopathic GnRH-dependent PP, 12 with PT, 3 with pathologic GnRH-dependent PP, and 3 with isolated menarche. Nineteen girls (13%) were adopted, of whom 15 had idiopathic GnRH-dependent PP, 3 had PT, and 1 had early menarche. None of the girls with PT had received the diagnoses of GnRH-dependent PP or early menarche after presenting with PT. Among the 6- to 8-year-old girls with PT, the age at menarche was available for 11 girls (31%). In all of them, menarche occurred within the normal limits for Finnish girls (5).

### Brain MRI Findings

A brain MRI was performed in 56 girls with GnRH-dependent PP, including all girls younger than 6 years of age and in 45 out of 66 girls aged 6–8 years (Table 2). Fourteen girls (25%) had an abnormal brain MRI and the findings were as follows: a hamartoma in five, other tumor in two (e.g., astrocytoma, optic glioma), craniopharyngioma in one, partial agenesis of the corpus callosum in one, hydrocephalus in one, pineal cyst in one, and other structural brain abnormalities unrelated to PP in three girls. Of the 14 abnormal MRI scans, five (three hamartomas, craniopharyngioma, and pineal cyst) were occult findings without any prior symptoms, signs or predisposing disease (Table S1 in Supplementary Material). Thus, the prevalence of an abnormal brain MRI in all girls without any predisposing disease was 3.6% (5/139) (95% CI 1.6–8.9%), and that in girls with GnRH-dependent PP was 6.4% (5/78) (95% CI 4.6–21.1%).

**Table 2:** MRI coverage in 78 girls with gonadotropin-releasing hormone-dependent precocious puberty (PP) aged 0.7–8 years.

Age (years)	<3	3–6	6–7	7–8
Proportion of girls with brain MRI scan available	4/4 (100%)	7/7 (100%)	7/12 (58%)	39/55 (71%)
Abnormal findings in MRI	2/4 (50%)	5/7 (71%)	2/7 (29%)	5/39 (13%)

*Patients are classified based on the age at presentation. Includes 10 girls with predisposing disease for PP*.

When the analysis was focused on the girls with GnRH-dependent PP aged 6–8 years and no predisposing disease for PP, a brain MRI was performed in 41 (68%) subjects (Figure 1B). An abnormal MRI was found in two girls (3.3%, 2/60) (95% CI 0.9–12.8%) (craniopharyngioma and pineal cyst) of which only the craniopharyngioma required immediate surgical treatment. Thus, in this subgroup, the prevalence of a CNS pathology requiring surgical intervention was 1.7% (1/60) (95% CI 0.3–9.7%), whereas it was 2.4% (1/41) (95% CI 0.4–14.4%) in girls who were assessed with a brain MRI. In those not imaged (*n* = 19), GnRH-dependent PP was followed closely in the outpatient clinic, and none of the girls presented any symptoms or signs of a lesion in CNS during the follow-up. All except one of the girls not imaged were more than 7 years old at presentation, and the majority of the patients were considered by the treating physician to have either borderline results in GnRH stimulation test (LH maximum 5–6 IU/L) or a feature that may increase the likelihood of idiopathic GnRH-dependent PP (adoption, positive family history of PP, or slow progression of puberty) or non-compliance to go to the scheduled brain MRI.

### Growth Velocity

In girls with PT (*n* = 39), the annual growth velocity was lower (7.2 ± 1.2 cm/year) and the bone age was less advanced (1.3 ± 1.1 years) than in the girls with GnRH-dependent PP (*n* = 71) (8.9 ± 2.3 cm/year, *P* < 0.001 and 2.0 ± 0.9 years, *P* < 0.001, respectively). The girls with GnRH-dependent PP had earlier age at take-off than the girls with PT (6.1 ± 1.4 vs 7.1 ± 0.9 years, *P* = 0.02). Interestingly, in girls with pathologic GnRH-dependent PP, the age at take-off occurred at younger age than in girls with idiopathic GnRH-dependent PP (4.9 ± 1.9 vs 6.4 ± 1.2 years, *P* = 0.003), but neither the growth velocity nor the advance in bone age differed from those with idiopathic GnRH-dependent PP (Table S2 in Supplementary Material).

Six- to eight-year-olds with GnRH-dependent PP grew faster than those with PT (8.7 ± 2.0 cm/year, *n* = 58 vs 7.0 ± 1.1 cm/year, *n* = 32, *P* < 0.001) and had more advanced bone age (2.0 ± 0.9 years, *n* = 51 vs 1.3 ± 1.1 years, *n* = 30, *P* = 0.002). Overall, growth velocity correlated positively with serum estradiol and GnRH-stimulated LH levels (*r* = 0.32, *P* = 0.003 and *r* = 0.56, *P* < 0.001, respectively), and faster growth velocity also increased the odds of GnRH-dependent PP (Table 3). In ROC analysis, the best growth velocity cut-off value, 7.0 cm/year, discriminated girls with GnRH-dependent PP (*n* = 58) from those with PT (*n* = 32) with a sensitivity of 92% and a specificity of 58% (AUC 0.82, 95% CI 0.73–0.91, *P* < 0.001). All girls with GnRH-dependent PP grew faster than 6 cm/year (sensitivity of 100% and specificity 21%), and none of those with PT grew faster than 9.2 cm/year (sensitivity of 25% and specificity of 100%).

**Table 3:** Predictors of GnRH-dependent PP in girls (*n* = 96) who presented with signs of puberty between ages 6 and 8 years and had no predisposing disease for PP.

	*N*	*n*	OR	95% CI	*P*-value
Age (years)	96	60	0.8	0.3–1.8	0.6
Growth velocity (cm/year)	90	58	2.9	1.7–4.9	<0.001
Bone age advancement (years)	81	51	2.1	1.3–3.5	0.005
Family history of PP	88	52			
No	57	31	1.0		
Yes	31	21	1.3	0.6–4.0	0.3
Adoption	96	59			
No	79	45	1.0		
Yes	17	14	3.3	0.9–12.7	0.08

*N, number of girls included in analysis; n, number of girls with GnRH dependent; PP, precocious puberty; OR, odds ratio; CI, confidence interval; GnRH, gonadotropin-releasing hormone*.

*All analyses were adjusted with age*.

## Discussion

We evaluated the etiology of PP in a large series of girls with a special focus on those aged 6–8 years, as their optimal management has been debated (9, 12, 14, 27). The challenges in the diagnostic work-up of PP stem from several sources, as the timing of puberty shows complex interactions between genetic predisposition, nutritional status, general health, environmental hormone-like compounds, and stress (28–32). In previously healthy asymptomatic 6- to 8-year-old girls, the prevalence of an abnormal brain MRI was 3.3%, and the prevalence of abnormal MRI requiring surgical intervention was 1.7%. Among the 6- to 8-year-olds, there were 19 girls who were not imaged, but none of them presented with signs of CNS processes during the follow-up which argues against significant CNS pathology. The 6.3-year-old girl with a craniopharyngioma had no prior symptoms or signs suggestive of a CNS lesion, and, thus, her diagnosis was expedited by current national clinical practice which recommends MRI imaging for girls who present with GnRH-dependent PP before the age of 8 years. Interestingly, this girl had a positive family history of PP, but overall in our cohort, the positive family history of early puberty was not a significant predictor of GnRH-dependent PP among 6- to 8-year-old girls. Recently, Giabicani et al. showed that girls evaluated for PP had more frequently pubertal LH/FSH ratio if they reported a family history of early puberty (33), but the study did not focus on girls aged 6–8 years.

The onset of breast development is the first sign of PP in girls. In obese girls, however, the presence of glandular breast tissue can be difficult to assess (12, 16), and if glandular tissue is present, the next task is to assess whether it is reactive due to increased estrogen levels or rather represents a local reaction of the hormone-sensitive tissue to low amounts of circulating estrogens. In the presence of low estrogen levels and prepubertal LH response in the GnRH stimulation test, and exclusion of peripheral puberty and exogenous sources of estrogens, the condition is termed PT. Expectedly, PT was the most frequent cause of PP in girls younger than 3 years, but it also explained a significant proportion (more than one-third) of the cases in 6- to 8-year-old girls presenting with PP, which poses a diagnostic challenge. Between 2001 and 2014, we found an increase in the number of girls diagnosed with PT but not in the frequency of girls with PT or with idiopathic GnRH-dependent PP, which may have resulted from the increased awareness of physicians referring girls with signs of PP, or from the possible influence of endocrine disruptors, or from a secular trend toward an earlier breast development. Supporting the latter, a recent Danish study reported a decline in the mean age of the appearance of glandular breast tissue without an earlier activation of the pituitary–gonadal axis (1). In our work, we defined PT as the presence of Tanner stage B2 before the age of 8 years, when the HPG axis was not activated according to the basal or GnRH-stimulated LH levels. However, it is possible that some of our patients who presented with PT represent slowly progressing variants of CPP. Altogether these findings suggest that earlier breast development results in an increase in the proportion of girls with PT evaluated by pediatric endocrinologists. However, our results need to be validated in a larger population-based cohort.

Besides breast tissue, another clinically useful cue for estrogen action is growth velocity, a sensitive marker of exposure to estrogen (19, 34). Estrogen is known to accelerate growth directly and through increased growth hormone secretion (35–37). Our study included extensive growth measurements enabling a thorough evaluation of growth curves and an accurate identification of the age at take-off. Although obesity is known to accelerate growth in childhood, and an increase of one BMI unit is associated with a height gain of 0.29 cm in girls (38), our data shows that growth velocity prior to the evaluation of PP was a strong predictor for GnRH-dependent PP and could be used in the differential diagnosis between PT and PP within this age group. In fact, growth velocity cut-off level of 7.0 cm/year identified effectively GnRH-dependent PP from PT in girls aged 6–8 years, and none of the girls with GnRH-dependent PP grew slower than 6 cm/year. In accordance with this finding, advanced bone age also emerged as a significant predictor of GnRH-dependent PP in 6- to 8-year-old girls. Although growth velocity overlapped between girls with PT and GnRH-dependent PP, the results validate the importance of careful evaluation of growth as part of the routine diagnostic process in girls with signs of early puberty.

The frequency of pathologic GnRH-dependent PP was more common in girls aged 3–6 years than in older girls. This is consistent with previous studies suggesting that young age clearly increases the risk of GnRH-dependent PP (8, 10, 39) and strongly supports the view that full diagnostic work-up, including brain MRI, is needed in this age group. The tendency toward an earlier onset of puberty is influenced by ethnicity. Furthermore, adopted girls are more likely to develop idiopathic GnRH-dependent PP than their native peers (7, 15), and in our series adoption increased the odds of GnRH-dependent PP with a borderline statistical significance.

While the main limitation of the study is its retrospective design, it should be noted that a prospective design is very difficult to conduct when describing the etiology of rare diseases such as PP (10, 18). Moreover, full diagnostic work-up of PP was not performed in all subjects, and, especially, 32% of 6- to 8-year-old girls with GnRH-dependent PP did not have a brain MRI performed. However, these girls were followed up closely, and none of them presented with signs or symptoms of CNS pathology. Finally, our series included mainly Caucasian girls which should be taken into account when interpreting the results in countries with higher proportion of other ethnicities.

In conclusion, we found that the risk of GnRH-dependent PP is associated with growth velocity in 6- to 8-year-old girls. In this age group, the prevalence of abnormal MRI requiring intervention in asymptomatic and previously healthy girls was low, but a lesion in CNS was present in one girl without any prior symptoms or signs. The results support the concept that brain MRI should be performed in girls with GnRH-dependent PP younger than 8 years of age.

## Author Contributions

TV, HH, LK, JH, and AT collected and interpreted the data and wrote the first version of the article. PM, MH, and TR designed and supervised the study and interpreted the results. All authors made contributions in writing the article and approved the final version.

## Conflict of Interest Statement

The authors declare that the research was conducted in the absence of any commercial or financial relationships that could be construed as a potential conflict of interest.
