# Supplementary material for: Precocious Puberty or Premature Thelarche: Analysis of a Large Patient Series in a Single Tertiary Center with Special Emphasis on 6- to 8-Year-Old Girls
Source: Front Endocrinol (Lausanne). 2017 Aug 23;8:213. doi: 10.3389/fendo.2017.00213 (PMC5572337; doi:10.3389/fendo.2017.00213)
Supplement: Supplementary file 1 [file Table_1.PDF]

Supplementary Table 1. The diagnoses of ten girls with precocious puberty (PP) who had a previously known disease predisposing to PP.

ID	Predisposing disease that can cause PP	Age at presentation (yrs)
1	Neurofibromatosis type 1	7.6
2	Optic glioma	4.6
3	Agenesis of the corpus callosum	6.8
4	Astrocytoma and neurofibromatosis type 1	7.2
5	Hydrocephalus	7.5
6	Previously diagnosed wide ischemic changes in CNS	5.6
7	Previously diagnosed wide hypoxic-ischemic changes in CNS	2.7
8	Perinatal asphyxia, tetraplegia, and epilepsy	7
9	Perinatal infection that caused severe mental retardation, blindness, tetraplegia, and epilepsy	7.1
10	Ventriculomegaly	7.5

PP, precocious puberty; CNS, central nervous system.
