# Supplementary material for: Precocious Puberty or Premature Thelarche: Analysis of a Large Patient Series in a Single Tertiary Center with Special Emphasis on 6- to 8-Year-Old Girls
Source: Front Endocrinol (Lausanne). 2017 Aug 23;8:213. doi: 10.3389/fendo.2017.00213 (PMC5572337; doi:10.3389/fendo.2017.00213)
Supplement: Supplementary file 2 [file Table_2.PDF]

Supplementary Table 2. Clinical characteristics of 149 girls with precocious puberty according to the diagnostic subgroups

	**Idiopathic GnRH-dependent PP (n=65)**	**Pathologic GnRH-dependent PP (n=13)**	**Premature thelarche (n=57)**	**GnRH-independent PP (n=5)**	**Early or isolated menarche (n=9)**
Height SDS	1.3 (1.2) (n=60)	1.1 (1.2) (n=11)	0.9 (1.5) (n=39)	0.8 (n=1)	1.4 (0.9) (n=9)
Weight-for-height (%)	6.1 (14.9) (n=60)	11.9 (19.3) (n=11)	7.3 (14.2) (n=39)	-3.8 (n=1)	21.2 (13.8) (n=9)
BMI (kg/m^2)	17.5 (2.5) (n=60)	17.7 (3.0) (n=11)	17.5 (2.3) (n=39)	15.2 (n=1)	21.4 (2.5) (n=9)
Growth velocity (cm/yr)	8.9 (2.1) (n=60)	8.9 (2.3) (n=11)	7.1 (0.9) (n=39)	10.4 (n=1)	7.9 (1.2) (n=9)
Delta height SDS	0.6 (0.5) (n=60)	0.6 (0.5) (n=11)	0.1 (0.2) (n=39)	0.7 (n=1)	0.6 (0.4) (n=9)
Annual delta height SDS	0.5 (0.4) (n=60)	0.5 (0.5) (n=11)	0.1 (0.2) (n=39)	0.7 (n=1)	0.3 (0.2) (n=9)
Height SDS at take off	0.7 (1.1) (n=46)	0.8 (1.4) (n=8)	0.8 (1.3) (n=13)	0.8 (n=1)	0.7 (1.0) (n=6)
Age at take-off (yrs)	6.4 (1.2) (n=47)	4.9 (1.9) (n=8)	7.1 (0.9) (n=13)	3.1 (n=1)	8.0 (1.1) (n=6)
BA - CA (yrs)	2.0 (0.9) (n=52)	2.2 (0.8) (n=11)	1.3 (1.1) (n=35)		2.2 (1.3) (n=9)

Mean ± SD. PP, precocious puberty; SDS, standard deviation score; BMI, body mass index; BA, bone age; CA, calendar age.
